# Monthly Prices of Substances Used in Pharmacy

**Published:** 1814-10

**Authors:** 


### *Monthly Prices of* Substances *used in* Pharmacy,

At Messrs. SELWAY and HENLEY's (Chemical and Pharmaceutical Laboratory,) 35, Upper Mary-le-bone Street, Portland Place.

| | | | | | |
|---|---|---|---|---|---|
| Acetum Scillæ ........... lb. | 2*s.* | 0*d.* | Antimonii Sulphuretum .... | 1 | 0 |
| Abietis Resina ............ | 2 | 3 | ———— Aurum. Sulph. .. | 6 | 0 |
| Acaciæ Gummi, from 2*s.* 3*d.* to | 3 | 6 | ———— Murias ........ | 3 | 0 |
| Acidum Aceticum .... cong. | 3 | 6 | Antimonium Tartarizatum .. | 5 | 0 |
| ——— Benzoicum ...... oz. | 6 | 0 | Anthemidis Flores, 1*s.* 9*d.* to | 2 | 0 |
| ——— Oxalicum ...... lb. | 18 | 0 | Aqua Cinnamomi vera .... | 18 | 0 |
| ——— Nitricum .......... | 4 | 6 | ——— Rosæ .............. | 5 | 6 |
| ——— Nitrosum ......... | 2 | 6 | Aliæ Aquæ, from 2*s.* 6*d.* to | 3 | 6 |
| ——— Muriaticum ........ | 0 | 9 | Arsenici Oxydum ........ lb. | 1 | 0 |
| ——— Sulphuricum ...... | 0 | 6 | Argenti Nitras ............ | 6 | 9 |
| —————— Atom... | 5 | 0 | Assafœtida Gummi-resina .. | 6 | 9 |
| Alcohol sp. gr. 815 M. lb. | 4 | 6 | Aurantii Cortex .......... | 2 | 6 |
| Æther sulphur. sp. gr. 789 | 9 | 0 | Balsamum Peruvianum...... | 33 | 0 |
| ——— rectificatus sp. gr. 744 | 11 | 0 | ——— Tolutanum ...... | 15 | 0 |
| Ærugo ............... lb. | 6 | 0 | Benzoinum elect. .......... | 11 | 0 |
| Aloes spicatæ extractum .... | 5 | 6 | Calamina præp. .......... | 0 | 3 |
| ——— vulgaris extractum .... | 4 | 6 | Calumbæ Radix .......... | 2 | 6 |
| ——— Barbadoes ........... | 5 | 0 | Cambogia opt. ............ | 9 | 6 |
| Alumen, 4*d.* exsiccatum.... | 0 | 8 | Camphora ............... | 6 | 6 |
| Ammoniæ Murias, 2*s.* 2*d.* to | 2 | 9 | Canellæ Cortex .......... | 3 | 4 |
| ——— Carbonas ........ | 3 | 6 | Cardamomi Semina opt..... | 12 | 0 |
| Amygdalæ Amaræ, 2*s.* Dulc. | 5 | 8 | Cascarillæ Cortex .......... | 4 | 0 |
| Ammoniacum, from 3*s.* 6*d.* to | 6 | 6 | Castoreum ............ unc. | 4 | 4 |

| | | |
|---|---|---|
| Caryophilli ... lb. | 11 | 0 |
| Cassiæ Cortex | 8 | 0 |
| Catechu Extractum | 2 | 3 |
| Cetaceum | 3 | 0 |
| Cera flava, 3*s*. 6*d*. alba | 3 | 6 |
| Cerata, from 2*s*. 6*d*. to | 4 | 0 |
| Cinchonæ cordif. Cort. | 9 | 0 |
| —— lancif. Cort. | 13 | 0 |
| —— oblongif. Cort. | 16 | 0 |
| Cinnamomi Cortex | 17 | 0 |
| Coccus (Coccinella) | 60 | 0 |
| Colocynthidis | 8 | 0 |
| Copaiba | 7 | 0 |
| Cornu usti | 1 | 0 |
| Cretæ ppt. | 0 | 8 |
| Croci stigmata | 64 | 0 |
| Cupri sulphas | 1 | 0 |
| Cuprum ammoniatum | 6 | 0 |
| Curcumæ Pulvis | 2 | 6 |
| Confectio Aromatica | 8 | 6 |
| —— Aurantii | 2 | 9 |
| —— Opii | 4 | 6 |
| —— Rosæ Gallicæ | 1 | 10 |
| —— Caninæ | 2 | 0 |
| —— Scammoniæ | 15 | 0 |
| —— Sennæ | 2 | 0 |
| —— Amygdalæ | 5 | 0 |
| Emplastrum Ammoniaci | 8 | 0 |
| —— Cumini | 2 | 0 |
| —— Galbani Comp. | 2 | 6 |
| —— Lyttæ | 6 | 6 |
| —— Opii | 2 | 6 |
| —— Plumbi | 1 | 6 |
| —— Resinæ | 1 | 6 |
| —— Roborans | 2 | 0 |
| —— Saponis | 2 | 6 |
| —— Hydrargyri | 2 | 6 |
| —— cum Amm. | 6 | 0 |
| Extractum Anthemidis .. unc. | 1 | 0 |
| —— Belladonnæ | 1 | 0 |
| —— Cinchonæ | 3 | 0 |
| —— resinos. | 4 | 0 |
| Extractum Colocynthidis | 2 | 0 |
| —— comp. | 1 | 0 |
| —— Conii | 0 | 6 |
| —— Gentianæ | 0 | 5 |
| —— Glycyrrhizæ .. lb. | 3 | 0 |
| —— Cascarillæ ... unc. | 3 | 0 |
| —— Hæmatoxyli | 0 | 5 |
| —— Humuli | 0 | 9 |
| —— Hyoscyami | 1 | 0 |
| —— Jalapæ 1*s*. 6*d* Res. | 2 | 8 |
| —— Opii aquos | 3 | 8 |
| —— resinos | 3 | 0 |
| —— Papaveris | 0 | 6 |
| —— Rhæi | 2 | 4 |
| —— Sarsaparillæ | 0 | 10 |
| —— Taraxaci | 0 | 6 |
| Ferri carbon. 1*s*. 0*d*. sulph. lb. | 1 | 0 |
| Ferrum Ammoniatum | 3 | 6 |
| —— Tartarizatum | 4 | 0 |
| Galbani Gummi-resinæ | 8 | 6 |
| Gentianæ Radix | 1 | 3 |
| Guaiaci Resina | 6 | 0 |
| Hydrargyrus purificatus | 5 | 4 |
| —— cm Creta | 4 | 0 |
| Hydrargyri Oxymurias | 8 | 0 |
| —— Submurias | 8 | 6 |
| —— Hydros. | 10 | 0 |
| —— Nitrico-Oxydum | 8 | 6 |
| —— Oxydum Rubrum | 8 | 0 |
| —— Sulphuretum Nig. | 3 | 6 |
| —— Præcipitatus Alb. | 8 | 0 |
| Hellebori nigri Radix | 2 | 6 |
| Ipecacuanhæ Radix | 22 | 0 |
| Jalapæ Radix | 6 | 0 |
| Kino | 12 | 0 |
| Liquor Plumbi Acetatis M. lb. | 1 | 0 |
| —— Ammoniæ .... P.L. | 2 | 8 |
| —— Vol. Corn. Cerv. | 1 | 3 |
| Linimentum Camphoræ comp. | 5 | 6 |
| —— Saponis comp. | 5 | 6 |
| Lichen Islandicus ... P. lb. | 1 | 9 |
| Lini Semines | 0 | 6 |
| Lyttæ | 10 | 0 |
| Magnesia | 8 | 6 |
| —— Carbonas | 3 | 0 |
| —— Sulphas | 1 | 2 |
| Manna optima | 6 | 0 |
| Mel Rosæ | 2 | 6 |
| Moschus pod, 24*s*. in gr. unc. | 40 | 0 |
| Mastiche ... lb. | 5 | 6 |
| Myristicæ Nuclei | 25 | 0 |
| Myrrha elect. | 6 | 6 |
| Opium (Turkey) | 26 | 0 |
| Oleum Amygdale ... lb. | 4 | 4 |
| —— Anisi ... unc. | 2 | 0 |
| —— Anthemidis | 5 | 0 |
| —— Cinnamomi Cassiæ | 8 | 0 |
| —— Caryophili | 5 | 0 |
| —— Cajuputi | 8 | 0 |
| —— Carui | 1 | 9 |
| —— Juniperi | 0 | 9 |
| —— Lavandulæ | 4 | 0 |
| —— Lini ... cong. | 8 | 6 |
| —— Menthæ piperitæ unc. | 4 | 0 |
| —— viridis | 3 | 0 |
| —— Olivæ, from 14*s*. 0*d*. to | 24 | 0 |
| —— Origani ... unc. | 2 | 0 |
| —— Pimentæ | 4 | 6 |
| —— Ricini, from 7*s*. 0*d*. to | 10 | 0 |
| —— Rosmarini ... unc. | 0 | 9 |
| —— Succini .. 2*s*. 4*d*. rect. | 2 | 6 |
| —— Sulphuretum ... lb. | 1 | 6 |
| —— Terebinthinæ rectif. | 2 | 0 |
| Oxymel Scillæ | 3 | 0 |
| Papaveris Capsulæ (per 100) | 2 | 4 |
| Plumbi Carbonas ... lb. | 0 | 8 |
| —— Superacetas | 2 | 2 |
| Potassa Fusa ... unc. | 0 | 6 |
| —— cum Calce | 1 | 6 |
| Potassæ Nitras ... lb. | 1 | 0 |
| —— Acetas | 8 | 0 |
| —— Subcarbonas | 1 | 2 |
| —— Carbonas | 4 | 0 |
| —— Sulphas | 1 | 0 |
| —— Sulphuretum | 1 | 6 |
| —— Tartras | 3 | 0 |

| | | |
|---|---|---|
| Potassæ Supertartras | 1 | 10 |
| Pilulæ Hydrargyri | 6 | 0 |
| ——— Aloes comp. | 8 | 0 |
| ——— cum Colocynth. | 16 | 0 |
| ——— Scillæ Compos. | 6 | 6 |
| ——— e Styrace | 48 | 0 |
| Piper longum et Nigrum | 2 | 6 |
| Pimento lb. | 2 | 4 |
| Pulvis Aloes cum Canella | 6 | 6 |
| ——— Compositus | 10 | 0 |
| ——— Antimonialis | 7 | 0 |
| ——— Cinnamomi Comp. | 16 | 0 |
| ——— Cornu cervi cum Opio | 14 | 0 |
| ——— Cretæ Composit. | 8 | 0 |
| ——— cum Opio | 8 | 0 |
| ——— Kino Compositus | 12 | 0 |
| ——— Scammoniæ Comp. unc. | 2 | 6 |
| ——— Sennæ Composit. lb. | 8 | 0 |
| ——— Tragacanthæ Comp. | 5 | 0 |
| Resina Flava et Nigra | 0 | 5 |
| Rosæ Folia | 7 | 6 |
| Ras Quassiæ | 1 | 6 |
| Rhæi Radix (Russia) | 30 | 0 |
| ——— (East India) | 14 | 0 |
| Sapo (Spanish) | 2 | 6 |
| Sarsaparilla Radix Inc. | 5 | 0 |
| Scammoniæ Gum-Resina unc. | 2 | 6 |
| Scilla recens, 1*s.* 3*d.* siccata | 5 | 0 |
| Sennæ Folia Opt. Alexand. | 6 | 6 |
| Serpentariæ Radix | 30 | 0 |
| Simaroubæ Cortex | 4 | 0 |
| Sodæ Boras | 4 | 6 |
| ——— Carbonas | 7 | 0 |
| ——— Subcarbonas | 1 | 4 |
| Sodæ Sulphas | 0 | 8 |
| ——— Phosphas | 2 | 8 |
| Soda tartarizata lb. | 2 | 4 |
| Spongia usta unc. | 1 | 3 |
| Spiritus Ammoniæ M. lb. | 3 | 6 |
| ——— aromaticus | 4 | 6 |
| ——— fœtidus | 3 | 6 |
| ——— succinatus | 4 | 6 |
| ——— Cinnamomi ver. | 3 | 6 |
| ——— Juniperi Compos. | 3 | 0 |
| ——— Lavandulæ | 4 | 6 |
| ——— Compos. | 5 | 0 |
| ——— Myristicæ | 3 | 0 |
| ——— Pimentæ | 3 | 0 |
| ——— Rosmarini | 3 | 0 |
| ——— Ætheris Aromaticus | 6 | 0 |
| ——— Compositus | 6 | 0 |
| ——— Nitrici sp. gr. 860 | 5 | 6 |
| ——— Sulphurici | 6 | 0 |
| ——— Vini rectificatus | 24 | 0 |
| Syrupi, from 1*s.* 8*d.* to | 3 | 0 |
| Sulphur | 0 | 8 |
| ——— Præcipitatum | 1 | 0 |
| Tamarindus opt. | 2 | 6 |
| Terebinthina Vulgaris | 0 | 9 |
| ——— Canadensis | 8 | 0 |
| ——— Chia | 6 | 0 |
| Tragacantha Gummi | 10 | 0 |

| | | |
|---|---|---|
| Tinctura Aurantii M. lb. | 3 | 9 |
| ——— Aloes | 4 | 0 |
| ——— Composita | 9 | 6 |
| ——— Assafœtidæ | 5 | 6 |
| ——— Balsa. Tolut. | 5 | 6 |
| ——— Benzoini Comp. | 6 | 9 |
| ——— Camphoræ Comp. | 4 | 0 |
| ——— Cardamomi | 4 | 0 |
| ——— Comp. | 4 | 0 |
| ——— Calumbæ | 3 | 9 |
| ——— Capsici | 3 | 9 |
| ——— Cascarillæ | 4 | 6 |
| ——— Castorei | 7 | 0 |
| ——— Catechu | 4 | 0 |
| ——— Cinchonæ | 6 | 0 |
| ——— Compos. | 6 | 0 |
| ——— Cinchonæ Ammon. | 7 | 0 |
| ——— Croci | 6 | 6 |
| ——— Digitalis | 4 | 6 |
| ——— Ferri Ammoniati | 4 | 0 |
| ——— Cinnamomi | 4 | 6 |
| ——— Comp. | 4 | 6 |
| ——— Gentianæ Comp. | 4 | 0 |
| ——— Guaiaci | 6 | 0 |
| ——— Ammon. | 6 | 0 |
| ——— Hellebori Nigri | 4 | 6 |
| ——— Humuli | 5 | 0 |
| ——— Hyoscyami Nig. | 4 | 6 |
| ——— Jalapæ | 4 | 6 |
| ——— Japonica | 4 | 6 |
| ——— Ferri Muriatis | 5 | 0 |
| ——— Kino | 5 | 0 |
| ——— Lyttæ | 3 | 9 |
| ——— Myrrhæ | 4 | 6 |
| ——— Opii | 7 | 0 |
| ——— Camphorat. | 4 | 6 |
| ——— Quassiæ | 3 | 9 |
| ——— Rhei | 4 | 6 |
| ——— Comp. | 4 | 0 |
| ——— Scillæ | 4 | 6 |
| ——— Sennæ | 4 | 9 |
| ——— Serpentariæ | 6 | 0 |
| ——— Valerianæ | 4 | 6 |
| ——— Amm. | 5 | 6 |
| ——— Zingiberis | 4 | 6 |
| Valerianæ Radix | 1 | 8 |
| Veratri Radix | 1 | 6 |
| Unguentum Hydrargyri fort | 5 | 0 |
| ——— Mit. | 3 | 0 |
| ——— Nitratis | 3 | 3 |
| ——— Nitrico-oxy. | 2 | 9 |
| ——— Sulphuris Comp. | 1 | 9 |
| ——— Alia ung. 1*s.* 8*d.* to | 2 | 0 |
| Uvæ Ursi Folia | 2 | 4 |
| Vinum Aloes | 3 | 6 |
| ——— Antimoniale | 3 | 0 |
| ——— Ferri | 3 | 6 |
| ——— Ipecacuanhæ | 5 | 6 |
| ——— Opii | 8 | 0 |
| Zinci Oxydum | 4 | 6 |
| ——— Sulphas purif. | 2 | 0 |
| Zingiberis Radix opt. | 2 | 4 |

**Leeches, 24s. per hundred.—Essential Salt of Lemons, 4s. 6d. per dozen.**

